# Correlation between bone metastasis and thrombocytosis in pulmonary adenocarcinoma patients

**DOI:** 10.3892/ol.2014.2770

**Published:** 2014-12-04

**Authors:** WEI ZHANG, CHAO YU, BIN HUANG, FENG-LIANG ZHOU, HAI-DONG HUANG, QIANG LI

**Affiliations:** 1 Department of Respiratory Medicine, Changhai Hospital, Second Military Medical University, Shanghai 200433, P.R. China; 2 Department of Respiratory Medicine, No. 413 Hospital of People’s Liberation Army, Zhoushan, Zhejiang 316000, P.R. China

**Keywords:** pulmonary adenocarcinoma, platelet count, bone metastasis

## Abstract

Thrombocytosis is commonly observed in patients exhibiting a variety of malignancies, including pulmonary, gastrointestinal and hepatic cancer. In the present study, the correlation between distant metastasis and thrombocytosis was retrospectively reviewed in 308 cases of histopathologically confirmed pulmonary adenocarcinoma. The patients were classified as having thrombocytosis or not, based on their platelet counts upon diagnosis; thrombocytosis was documented in 82/308 patients (26.6%). A log-rank test indicated a statistically significant difference in survival between patients exhibiting thrombocytosis compared with patients not exhibiting thrombocytosis (P<0.001). In addition, the occurrence of distant metastasis and the survival period were correlated with the presence of thrombocytosis upon diagnosis. In descending order of frequency, metastases were documented at the following sites: Lymph nodes (218/308 patients; 70.8%), bone (138/308 patients; 44.8%), lung (93/308 patients; 30.2%), brain (67/308 patients; 21.8%), liver (46/308 patients; 4.9%), adrenal glands (11/308 patients; 3.6%) and kidneys (5/308 patients; 1.6%). Bone metastasis occurred significantly more frequently in patients exhibiting thrombocytosis (50/82 patients: 61.0%; P<0.05) compared with patients not exhibiting thrombocytosis (88/226 patients; 38.9%). Furthermore, according to univariate analysis, thrombocytosis, weight loss, an Eastern Cooperative Oncology Group performance status score of ≥2 points, anemia, increased erythrocyte sedimentation rate, and increased alkaline phosphatase (AKP) and carcinoembryonic protein (CEA) levels were risk factors for bone metastasis. According to multivariate analysis, thrombocytosis, weight loss, and increased AKP and CEA levels were correlated with bone metastasis. Therefore, patients exhibiting pulmonary adenocarcinoma and thrombocytosis have a higher risk of bone metastasis compared with patients not exhibiting thrombocytosis.

## Introduction

Thrombocytosis is commonly observed in patients exhibiting malignant tumors. Retrospective studies have demonstrated that 10–60% of patients with untreated malignancies, such as pulmonary, gastrointestinal and hepatic cancers (1–3), exhibit increased platelet (PLT) counts. The frequency of associated thrombocytosis in primary lung cancer patients is ~16–32% and PLT counts range from 350×10^9^/l to 1,000×10^9^/l, or >1,000×10^9^/l in rare cases (4–6). Previous investigations have proposed that thrombocytosis is an independent predictor of poor prognosis in patients exhibiting malignancies, including primary lung cancer (7,8).

The mechanism(s) underlying the association between thrombocytosis and malignancy remains unknown. As demonstrated in previous studies, increased concentrations of humoral factors, such as thrombopoietin, interleukin-6 and interleukin-11, stimulate PLT production in patients exhibiting malignancies (9–11). In addition, the bone marrow microenvironment (12), PLT granule protein (13) and coagulation system activation may be important in the development of reactive thrombocytosis (14).

The mechanism(s) responsible for the poor prognosis of patients exhibiting malignant tumors and concomitant thrombocytosis requires elucidation. As demonstrated by a previous study, PLT aggregation induced by tumor cells contributes to the adhesion and encapsulation of PLTs with circulating tumor cells (15). This enhances the ability of tumor cells to escape the destructive effects of immune surveillance cells, such as natural killer cells. Furthermore, tumor cell-induced PLT aggregation may promote microcirculatory adhesion and colonization of tumor cells; thus, PLTs are involved in the development of hematogenous metastases (16). In addition, activated PLTs release vascular endothelial growth factor, epidermal growth factor, PLT-derived growth factor and a number other cytokines, which stimulate the growth of malignant cells and promote angiogenesis (17). PLTs are also involved in the development of Trousseau syndrome (18); however, it is not known whether there is a statistically significant difference in PLT activation and its effects on prognosis between patients with and without thrombocytosis, and whether any such differences are clinically relevant. Previous clinical studies have provided no consistent evidence to support a correlation between the incidence of thrombocytosis, and tumor node metastasis (TNM) stage, differentiation or tumor size. Furthermore, according to the majority of studies (1,3), the incidence of thrombocytosis is independent of the tumor pathology. Thus, the mechanism(s) responsible for the poor prognosis of patients exhibiting malignancies and concomitant thrombocytosis requires further investigation.

In the present study, the clinical data of 308 pulmonary adenocarcinoma patients were retrospectively analyzed and correlations between thrombocytosis and clinicopathological features, prognosis and distant metastases (particularly to the bone) were investigated.

## Patients and methods

### Patients

The records of 758 patients with histopathologically confirmed pulmonary adenocarcinoma, admitted to Changhai Hospital (Shanghai, China) from 1 July 2006 to 30 April 2009, were assessed in the present study. Patients exhibiting other tumors (previous and current), and blood, rheumatic, acute and chronic infectious or chronic inflammatory diseases, were excluded. Thus, 523 patients were selected for follow-up by telephone to obtain information regarding survival periods and distant metastasis. The median survival period was 93.9 weeks (range, 3.6–299.0 weeks) for 308/523 patients; the remaining 215 patients were lost to follow-up as they could not be contacted. The following data was collected from the study patients: i) General data, including gender, age and smoking index; and b) post-diagnostic data, including Eastern Cooperative Oncology Group performance status (ECOG PS) score (19), TNM stage (TNM, 6th edition) (20), white blood cell (WBC) count, hemoglobin, albumin, carcinoembryonic antigen (CEA) and alkaline phosphatase (AKP) levels, PLT count, erythrocyte sedimentation rate (ESR), activated partial thromboplastin time, tumor differentiation and metastasis. The overall survival (OS) was defined as the time in weeks from definite diagnosis to all-cause mortality or the termination of follow-up (30 June, 2012). Written informed consent was obtained from all patients.

### Measurements and PLT counts

The date of definite diagnosis was defined as the date the Department of Pathology, Changhai Hospital received samples of the tumor. Survival was defined as the time (in weeks) from the date of definite diagnosis to all-cause mortality or the cut-off date of 30 June, 2012. Peripheral blood PLT counts were measured using an ADVIA^®^ 120 (Siemens AG, Erlangen, Germany) hematology analyzer.

### Statistical analysis

Data were analyzed using SPSS software (version 17.0; SPSS Inc., Chicago, IL, USA). The normality of the PLT count distribution was determined by performing a Kolmogorov-Smirnov test and the association between thrombocytosis and tumor pathology was analyzed using univariate and multivariate analyses. The Wald test was also performed. The Cox proportional-hazards regression model, the last observation carried forward and the Kaplan-Meier method were employed for survival analysis. In addition, inter-group comparisons of survival were based on the log-rank test and inter-group comparisons of remote metastasis were performed using the χ^2^ test. P<0.05 was considered to indicate a statistically significant difference.

## Results

### General data

**Figure 1:**
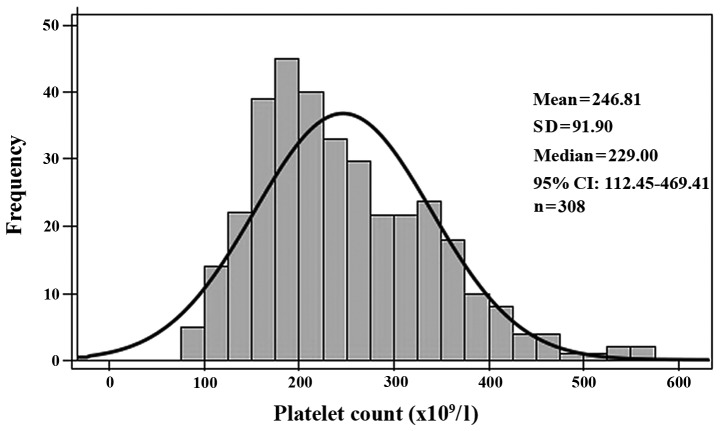
Peripheral blood platelet counts of 308 pulmonary adenocarcinoma patients are right-skewed (Kolmogorov-Smirnov test, Z=1.599; two-sided P-value, 0.012). SD, standard deviation; CI, confidence interval.

The present study included 308 pulmonary adenocarcinoma patients, aged 27–83 years (mean age ± standard deviation, 59.6±10.3). The gender ratio was 2.2:1 (213 male patients; 95 female patients) and TNM stage varied as follows: Stage I, 71 cases; Stage II, 32 cases; Stage IIIa, 44 cases; Stage IIIb, 49 cases; and Stage IV, 112 cases. Upon diagnosis, the mean PLT count was 246.8±91.9×10^9^/l; this included four (1.3%), 222 (72.1%) and 82 (26.6%) patients with PLT counts below, within and above the normal range (100–300×10^9^/l), respectively. A right-skewed distribution of PLT counts was observed (Fig. 1).

### Clinicopathological characteristics

The study patients were classified as having thrombocytosis (PLT≥300×10^9^/l; 82 cases) or not (PLT<300×10^9^/l; 226 cases). This factor, together with various other clinicopathological factors, was subjected to univariate analysis, which revealed that an ECOG PS score of ≥2 points, advanced TNM stage and leukocytosis were risk factors for thrombocytosis (Table I). According to multivariate analysis, leukocytosis, anemia and increased ESR were correlated with thrombocytosis (Table II).

### Overall survival and survival rate

**Figure 2:**
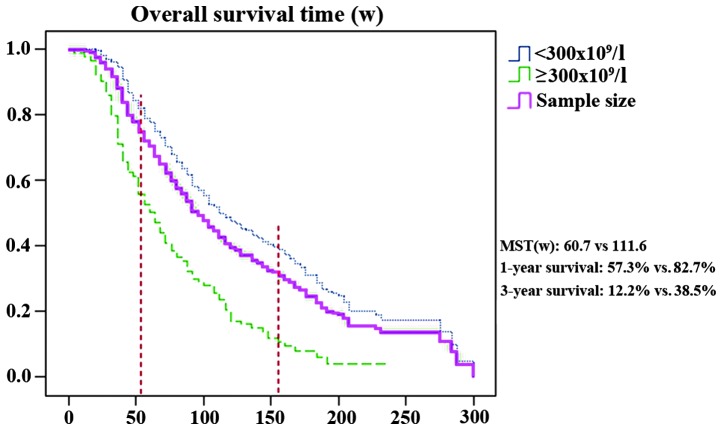
Survival was significantly poorer in patients exhibiting thrombocytosis compared with patients not exhibiting thrombocytosis (P<0.001). w, weeks; MST, mean survival time.

By final follow-up, 244/308 study patients had died and 64 study patients had survived. The one- and three-year survival rates were 76.0 and 31.5%, respectively. The 82 thrombocytosis patients had a mean survival time (MST) of 60.7 weeks (range, 3.6–235.9); however, the 226 non-thrombocytosis patients had an MST of 111.6 weeks (range, 16.6–299.0). Inter-group differences between one- and three-year survival rates (P<0.001; Table III) and within overall survival (log rank, χ^2^=43.095; P<0.001; Fig. 2) were statistically significant.

### Bone metastasis and thrombocytosis

Bone metastases were identified upon diagnosis in 29 (35.4%) and 51 (22.6%) patients with and without thrombocytosis, respectively. This inter-group difference was statistically significant (χ^2^=5.127; P=0.024). Of the thrombocytosis patients, 50 (61.0%) developed bone metastases during the course of disease progression, in comparison with 88 (38.9%) non-thrombocytosis patients. This difference was also statistically significant (χ^2^=11.816; P=0.001). However, differences in metastasis to sites other than bone were not statistically significant between thrombocytosis and non-thrombocytosis patients (Table IV). According to univariate analysis, thrombocytosis, weight loss, an ECOG PS score of ≥2 points, anemia, increased ESR, and increased AKP and CEA levels were risk factors for bone metastasis (Table V). According to multivariate analysis, thrombocytosis, weight loss, and increased AKP and CEA levels were correlated with bone metastasis (Table VI).

## Discussion

Thrombocytosis can be divided into two major categories: Clonal thrombocytosis and reactive thrombocytosis. Clonal thrombocytosis is induced by clonal myeloproliferative diseases, including idiopathic thrombocythemia and polycythemia vera (11). By contrast, reactive thrombocytosis is a secondary response to various factors, including infection, cancer and tissue injury, and is the most common type of thrombocytosis. Reactive thrombocytosis has been observed in various malignancies, including lung, gastrointestinal tract and liver cancer, at a reported incidence rate of 10–60% (1–3). In the present study, the clinical data of 308 pulmonary adenocarcinoma patients was retrospectively analyzed. The clinical characteristics of patients exhibiting thrombocytosis and pulmonary adenocarcinoma, as well as correlations between thrombocytosis and various clinicopathological factors, were investigated and are discussed from a clinical perspective.

In the current study, the incidence of thrombocytosis was 26.6%, which is consistent with the 16–32% reported in studies from other countries (1–3). A PLT count of ≥300×10^9^/l was chosen as the criterion for the diagnosis of thrombocytosis. Peripheral blood PLT counts exhibited a right-skewed distribution, indicating that untreated pulmonary adenocarcinoma patients have higher PLT counts than healthy subjects. Thus, the present study identified the phenomenon of pulmonary adenocarcinoma-associated thrombocytosis in Chinese patients. A PLT count of ≥300×10^9^/l was selected as the criterion for diagnosing thrombocytosis, as it defines the upper limit of the 95% CI for PLT counts in healthy Chinese subjects. By contrast, the majority of reports from other countries use a PLT count of ≥400×10^9^/l or ≥350×10^9^/l as the criterion for thrombocytosis. This difference in the normal range of PLT counts between Europe and the USA, and China, must be addressed; it may be associated with differences in ethnicity and/or measurement instruments. Using pairwise comparison, Zeng *et al* (21) identified that the frequency of the thrombopoietin receptor (TPOR) C allele at position 550 is significantly higher in subjects with high PLT counts, and that thrombocytosis is associated with a C to A transversion at position 550 in the 5′-promoter region of TPOR (21). Therefore, the present study postulates that genetic factors may be involved in the mechanism that determines the differences in the normal range of PLT in counts in Chinese subjects versus European and American subjects.

Previous studies have found that advanced TNM stage, a high ECOG PS score and poorly differentiated carcinomas are associated with poor prognosis in lung carcinoma (22). In the present study, univariate analysis demonstrated that an ECOG PS score of ≥2 points, advanced TNM staging and leukocytosis were risk factors for decreased overall survival. Multivariate analysis demonstrated that thrombocytosis is an independent risk factor for poor prognosis in pulmonary adenocarcinoma, with a relative risk of 2.103–3.814, indicating that the mortality of pulmonary adenocarcinoma with thrombocytosis is 2.103–3.814-fold greater than non-thrombocytosis patients. Furthermore, thrombocytosis patients exhibit a significantly shorter MST compared with non-thrombocytosis patients (difference, 50.9 weeks; 60.7 vs.111.6 weeks). In addition, the one- and three-year survival rates were significantly lower compared with patients not exhibiting thrombocytosis (difference, 25.4%; 57.3 vs. 82.7% and difference, 26.3%, 12.2 vs. 38.5%, respectively). Therefore, pulmonary adenocarcinoma patients exhibiting thrombocytosis have a worse prognosis than patients not exhibiting thrombocytosis, which is consistent with the findings reported for other malignancies.

Numerous reports from other countries support the significant effects of thrombocytosis on the survival of patients exhibiting malignancies (23–25). Thrombocytosis is a common presentation of paraneoplastic syndrome and has been recognized to accompany the development, proliferation, differentiation, invasion and metastasis of specific tumors. Furthermore, thrombocytosis may effect the survival of patients exhibiting various malignancies. Besides the stimulatory effects of activated and increased PLTs on hematogenous metastasis, tumor growth, and angiogenesis, the presence of thrombocytosis may correlate with the biological characteristics and behavior of malignant cells. Malignancies with associated thrombocytosis may possess different characteristics with regard to differentiation, invasion and metastasis compared with the same types of malignancy not associated with thrombocytosis. Furthermore, interaction of these factors may affect the patients’ prognoses (26–28).

Distant metastasis is an important biological characteristic of malignant tumors and an important prognostic factor (29). In the present study, pulmonary adenocarcinoma most frequently metastasized, in descending order, to the lymph nodes, bone, lung, brain, liver, adrenal glands and kidney. Thus, the most common site of hematogenous metastases was the bone. As PLTs are produced by bone marrow, the present study assessed the correlation between PLT count and bone metastasis and identified a statistically significant correlation. The risk of bone metastasis in patients exhibiting pulmonary adenocarcinoma and thrombocytosis was 1.436-fold higher than in patients not exhibiting thrombocytosis. However, the correlation coefficient for thrombocytosis versus bone metastasis was weak. Possible explanations for the weakness of this correlation include the following: i) Reactive thrombocytosis may have been induced by a number of other factors, such as infection, rheumatic autoimmune disease and coronary heart disease (30,31); b) relevant data may not have been recorded during follow-up; c) thrombocytosis may only correlate with one type of bone metastasis (osteoblastic, osteolytic or mixed) (32); and d) the small sample size may have effected the sampling error and therefore the overall findings of the study.

The present report was a retrospective study, thus, perspective and randomized sampling design were not considered and substantial relevant data was unavailable due to various factors, such as incomplete records, incomprehensible data and non-uniform reporting of data. These factors may have compromised the findings of the present study. Therefore, larger studies of tumor-induced thrombocytosis should be conducted to clarify the findings of the present report.

In conclusion, the present retrospective study of 308 pulmonary adenocarcinoma patients identified that thrombocytosis correlates with the development of bone metastases.

## Figures and Tables

**Table I:** Univariate analysis of relevant clinicopathological risk factors for thrombocytosis.

	Frequency, n (%)					
					
Clinicopathological factor	Thrombocytosis	No thrombocytosis	OR	95% CI	χ^2^ value	P-value
ECOG PS score						
≤1	72 (87.8)	217 (96.0)				
≥2	10 (12.2)	9 (4.0)	3.213	1.262–8.180	7.011	0.008
TMN stage						
IA–IIIA	29 (35.4)	118 (52.2)				
IIIA–IV	53 (64.6)	108 (47.8)	1.231	1.012–2.094	6.845	0.009
Hematological factors						
WBC, ×10^9^/l						
<10.0	61 (74.4)	217 (96.0)				
≥10.0	21 (25.6)	9 (4.0)	7.406	3.351–16.367	32.012	<0.001
Hgb, g/l						
<120	26 (31.7)	42 (18.6)				
≥120	56 (68.3)	184 (81.4)	0.420	0.246–0.719	6.023	0.014
Albumin, g/l						
<30	48 (58.5)	177 (78.3)				
≥30	34 (41.5)	49 (21.7)	2.393	1.427–4.013	11.961	0.001
ESR, mm/H (n=242)						
<20	18 (29.5)	88 (48.6)				
≥20	43 (70.5)	93 (51.4)	2.741	1.609–4.670	10.835	0.001
APTT, sec (n=258)						
23–43	64 (91.4)	184 (97.9)				
>43	6 (8.6)	4 (2.1)	4.985	1.424–17.450	5.894	0.015
AKP, U/l						
<92	53 (64.6)	174 (77.0)				
≥92	29 (35.4)	52 (23.0)	1.896	1.129–3.184	4.740	0.029

OR, odds ratio; CI, confidence interval; ECOG PS, Eastern Cooperative Oncology Group performance status; TMN, tumor node metastasis; WBC, white blood cell; Hgb, hemoglobin; ESR, erythrocyte sedimentation rate; APPT, activated partial thromboplastin time; AKP, alkaline phosphatase.

**Table II:** Multivariate analysis of relevant clinicopathological risk factors for thrombocytosis (n=228).

Risk factor	OR	95% confidence interval	Wald value	P-value
Fever				
No				
Yes	2.575	1.098–6.039	4.365	0.030
WBC, ×10^9^/l				
<10.0				
≥10.0	7.596	2.997–19.255	9.941	0.002
Hgb, g/l				
≥120				
<120	3.360	1.376–4.735	6.417	0.011
Albumin, g/l				
<30				
≥30	2.543	1.262–5.124	5.662	0.017
ESR, mm/H				
<20				
≥20	2.323	1.194–4.517	6.215	0.013
APTT, sec				
≤43				
>43	7.869	1.917–32.301	8.273	0.004

OR, odds ratio; WBC, white blood cell; Hgb, hemoglobin; ESR, erythrocyte sedimentation rate; APPT, activated partial thromboplastin time.

**Table III:** Comparison of survival between thrombocytosis and non-thrombocytosis patients.

Prognosis	Total, n=308	Thrombocytosis, n=82	No thrombocytosis, n=226	χ^2^	P-value
Outcome, n (%)					
Survival	64 (2.8)	6 (7.3)	58 (25.7)		
Mortality	244 (79.2)	76 (92.7)	168 (74.3)	13.536	<0.001
Prognosis					
MST, weeks	94.0	60.7	111.6		
One-year survival, n (%)	234 (76.0)	47 (57.3)	187 (82.7)	21.310	<0.001
Three-year survival, n (%)	97 (31.5)	10 (12.2)	87 (38.5)	19.291	<0.001

MST, median survival time.

**Table IV:** Comparison of frequency of distant metastasis between thrombocytosis and non-thrombocytosis patients.

Distant metastasis site	Sample size, n (%) (n=308)	Thrombocytosis, n (%) (n=82)	No thrombocytosis, n (%) (n=226)	χ^2^	P-value
Lymph node					
Upon diagnosis	218 (70.8)	61 (74.4)	157 (69.5)	0.705	0.401
During the disease course	209 (67.9)	59 (72.0)	150 (66.4)	0.859	0.354
Bone					
Upon diagnosis	80 (26.0)	29 (35.4)	51 (22.6)	5.127	0.024
During the disease course	138 (44.8)	50 (61.0)	88 (38.9)	11.816	0.001
Lung					
Upon diagnosis	54 (17.5)	17 (20.7)	37 (16.4)	0.791	0.374
During the disease course	93 (30.2)	27 (32.9)	66 (29.2)	0.396	0.529
Brain					
Upon diagnosis	24 (7.8)	7 (8.5)	17 (7.5)	0.086	0.769
During the disease course	67 (21.8)	17 (20.7)	50 (22.1)	0.069	0.794
Liver					
Upon diagnosis	24 (7.8)	7 (8.5)	17 (7.5)	0.086	0.769
During the disease course	46 (14.9)	13 (15.9)	33 (14.6)	0.074	0.785
Adrenal gland					
Upon diagnosis	6 (1.9)	1 (1.2)	5 (2.2)	0.311	0.577
During the disease course	11 (3.6)	3 (3.7)	8 (3.7)	0.002	0.960
Kidney					
Upon diagnosis	2 (0.6)	1 (1.2)	1 (0.4)	0.563	0.453
During the disease course	5 (1.6)	3 (3.7)	2 (0.9)	2.898	0.089

**Table V:** Univariate analysis of relevant clinicopathological risk factors for bone metastasis (n=308).

	Frequency (%)					
					
Risk factor	Bone metastasis	No bone metastasis	OR	95% CI	χ^2^	P-value
Weight loss (n=288)						
Yes	33 (41.3)	39 (17.1)				
No	47 (58.5)	189 (82.9)	3.403	1.938–5.975	19.274	<0.001
ECOG PS score						
≥2	11 (13.8)	8 (3.5)				
≤1	69 (86.3)	220 (96.5)	4.384	1.695–11.336	10.731	0.001
PLT, ×10^9^/l						
≥300	29 (36.3)	53 (23.2)				
<300	51 (63.8)	175 (76.8)	1.878	1.084–3.253	8.127	0.008
Hgb, g/l						
<120	25 (31.3)	43 (18.9)				
≥120	55 (68.8)	185 (81.1)	0.511	0.287–0.911	5.285	0.022
ESR, mm/h (n=242)						
≥20	38 (68.7)	98 (53.9)				
<20	20 (31.3)	86 (46.1)	1.169	1.027–1.330	5.320	0.021
AKP, U/l						
≥92	39 (48.8)	42 (18.4)				
<92	41 (51.3)	186 (81.6)	1.580	1.270–1.966	28.152	<0.001
CEA, U/l (n=287)						
≥10	49 (63.8)	63 (30.7)				
<10	27 (36.3)	148 (69.3)	3.969	2.323–6.782	27.117	<0.001

OR, odds ratio; CI, confidence interval; ECOG PS, Eastern Cooperative Oncology Group performance status; PLT, platelet; Hgb, hemoglobin; ESR, erythrocyte sedimentation rate; AKP, alkaline phosphatase; CEA, carcinoembryonic protein.

**Table VI:** Multivariate analysis of relevant clinicopathological risk factors for bone metastasis (n=237).

Risk factor	OR	95% CI	Wald value	P-value
Weight loss				
No				
Yes	3.002	1.603–5.623	11.790	0.001
PLT, ×10^9^/l				
<300				
≥300	1.436	1.043–2.871	4.013	0.048
AKP, U/l				
<92				
≥92	3.466	1.887–6.364	16.068	<0.001
CEA, U/l				
<10				
≥10	2.916	1.621–5.247	12.751	<0.001

OR, odds ratio; CI, confidence interval; PLT, platelet; AKP, alkaline phosphatase; CEA, carcinoembryonic protein.
